# Compressed Sensing MR Image Reconstruction Exploiting TGV and Wavelet Sparsity

**DOI:** 10.1155/2014/958671

**Published:** 2014-10-13

**Authors:** Di Zhao, Huiqian Du, Yu Han, Wenbo Mei

**Affiliations:** School of Information and Electronics, Beijing Institute of Technology, Beijing 100081, China

## Abstract

Compressed sensing (CS) based methods make it possible to reconstruct magnetic resonance (MR) images from undersampled measurements, which is known as CS-MRI. The reference-driven CS-MRI reconstruction schemes can further decrease the sampling ratio by exploiting the sparsity of the difference image between the target and the reference MR images in pixel domain. Unfortunately existing methods do not work well given that contrast changes are incorrectly estimated or motion compensation is inaccurate. In this paper, we propose to reconstruct MR images by utilizing the sparsity of the difference image between the target and the motion-compensated reference images in wavelet transform and gradient domains. The idea is attractive because it requires neither the estimation of the contrast changes nor multiple times motion compensations. In addition, we apply total generalized variation (TGV) regularization to eliminate the staircasing artifacts caused by conventional total variation (TV). Fast composite splitting algorithm (FCSA) is used to solve the proposed reconstruction problem in order to improve computational efficiency. Experimental results demonstrate that the proposed method can not only reduce the computational cost but also decrease sampling ratio or improve the reconstruction quality alternatively.

## 1. Introduction

Magnetic resonance imaging (MRI) plays an important role in the field of medical diagnostics. Speeding up the scanning time has always been of interest to the MRI research community. Recently, compressed sensing (CS) theory [1–6] has been claimed to be able to achieve accurate reconstruction of a sparse or compressible signal from highly undersampled measurements. Applying CS to MRI can significantly reduce the scanning time without degrading the image quality [7–10]. These methods do not use extra prior information besides sparsity (or compressibility). In many practical MRI applications, a high resolution reference image with similar anatomical structure to the target image can be acquired easily. If we exploit the reference image to get more prior information, the sampling ratio can be further decreased. Ji and Lang [11] have demonstrated the possibility of using the subtraction of a prescanned high resolution reference image to improve sparsity for dynamic MRI. Majumdar et al. [12] propose an online MR image reconstruction method which assumes the difference image between the target and the reference images is sparse in pixel domain and utilizes the *l*_*p*_-norm (0 < *p* < 1) of the difference image as part of the cost function. Image contrast changes are not considered in the above-mentioned methods. Peng et al. [13] use a low-order generalized series model [14], a partial set of wavelets based on the prior information provided by the reference image to depict the global and local contrast changes, which improve the pixel domain sparsity of the difference image. These works ignore motion effects, even though motion effects between different scans widely exist in practice. The misalignment between image features caused by motion can significantly degrade the quality of the reconstructed images. Jung et al. [15–17] decompose the unknown spectral signal into the predicted signal and the residual signal and apply the generalized series model or motion estimation/compensation (ME/MC) to sparsify the residual signal. Methods proposed in [18, 19] model the target image as a linear combination of a motion-dependent reference image and a difference image where the affine transformation is used to compensate the motion effects and a scaling constant or generalized series model is introduced to sparsify the difference image in pixel domain.

In this paper, we model the target image as a sum of the motion-compensated reference image and the difference image. Our idea is based on the fact that contrast changes severely degrade the sparsity of the difference image in pixel domain but have little influence on the sparsity in wavelet transform and gradient domains. In addition, we find that motion effects degrade the sparsity in the above three domains, most severely in pixel domain. Hence, we propose to do motion compensation once at first and then apply wavelet transform and total generalized variation (TGV) [20–22] to sparsify the difference image. Second-order TGV, which involves second-order derivatives of an image, is used here to eliminate the staircasing artifacts caused by total variation (TV). The proposed method avoids the iterative estimation of the motion effects and improves the reconstruction efficiency. An efficient algorithm named as fast composite splitting algorithm (FCSA) [23, 24] is employed to solve the proposed reconstruction problem. We evaluate the proposed method by conducting experiments on three sets of practical MRI data. The experimental results demonstrate that the proposed method outperforms conventional CS-MRI methods and the pixel-sparsity based reference-driven method.

The rest of this paper is organized as follows.  Section 2 describes the proposed method in detail. The analysis on how contrast changes and motion effects affect the sparsity of the difference image is also included.  Section 3 shows experimental results from three sets of real MR data.  Section 4 discusses relevant properties of the proposed method. Finally, conclusions are drawn in Section 5.

## 2. Proposed Method

### 2.1. Image Model

In many practical MRI applications, a high resolution reference image with similar anatomical structure to the target image can be acquired easily. We express the target image **I**_**t**_(**r**) as follows:

(1) It(r)=Ir(r)+Id(r),

where **I**_**r**_(**r**) is the reference image and **I**_**d**_(**r**) is the difference image.

Considering motion effects existing between **I**_**t**_(**r**) and **I**_**r**_(**r**), we rewrite (1) as

(2) $$\begin{array}{c} I_{t}\left(r\right)=I_{r}\left(T\left(r\right)\right)+I_{d}\left(r\right), \end{array}$$

where **I**_**r**_(**T**(**r**)) is a deformable reference image depending on the coordinate transformation **T**(**r**).

The data acquisition is formulated as

(3) $$\begin{array}{c} d_{t}=F_{u}\left(I_{r}\left(T\right)+I_{d}\right)+n, \end{array}$$

where **d**_**t**_ is the measured data vector of the target image, **F**_**u**_ is an undersampled Fourier transform operator, and **n** is the measurement noise.

In this paper, we apply an affine transformation **T** to characterize the global motion effects between the target and the reference images. We employ the method in [18] to estimate affine motion parameters by solving the optimization problem

(4) $$\begin{array}{c} \hat{T}=\arg\underset{T}{\min}{||d_{t}-F_{u}(I_{r}(T))||}_{2}^{2}. \end{array}$$

The Broyden-Fletcher-Goldfarb-Shanno (BFGS) algorithm is used to estimate the affine parameters in (4). The local deformation between the target and the reference images can be regarded as part of the difference image. Such approach avoids the complex local motion estimation used in [25–27].

### 2.2. Reconstruction Model

In our proposed method, the reconstruction is modeled as

(5) $$\begin{array}{c} {\hat{I}}_{d}=\arg\underset{I_{d}}{\min}{||d_{t}-F_{u}\left({\tilde{I}}_{r}+I_{d}\right)||}_{2}^{2}+\lambda ||\Psi I_{d}{||}_{1}+\text{TG}{{\text{V}}_{\alpha }}^{2}\left(I_{d}\right), \end{array}$$

where ${\hat{I}}_{d}$ denotes the estimation for the difference image **I**_**d**_ and the motion-compensated reference image is

(6) $$\begin{array}{c} {\tilde{I}}_{r}=I_{r}\left(\hat{T}\right). \end{array}$$

Here, $\hat{T}$ is the estimation of the transformation **T** from (4). The linear operator Ψ is the wavelet transform operator. *λ* is a positive regularization parameter. TGV_*α*_^2^(·) denotes the second-order TGV operator, which combines the first and second derivatives to yield discrete gradients of an image.

#### 2.2.1. Explanations to the Reconstruction Model

The two regularization terms in the objective function equation (5) enforce the sparsity of the difference image in wavelet transform and gradient domains. It is based on the fact that contrast changes and motion effects severely degrade the sparsity of the difference image in the pixel domain but have little influence on the sparsity in the wavelet transform and gradient domains.

**Figure 1:**
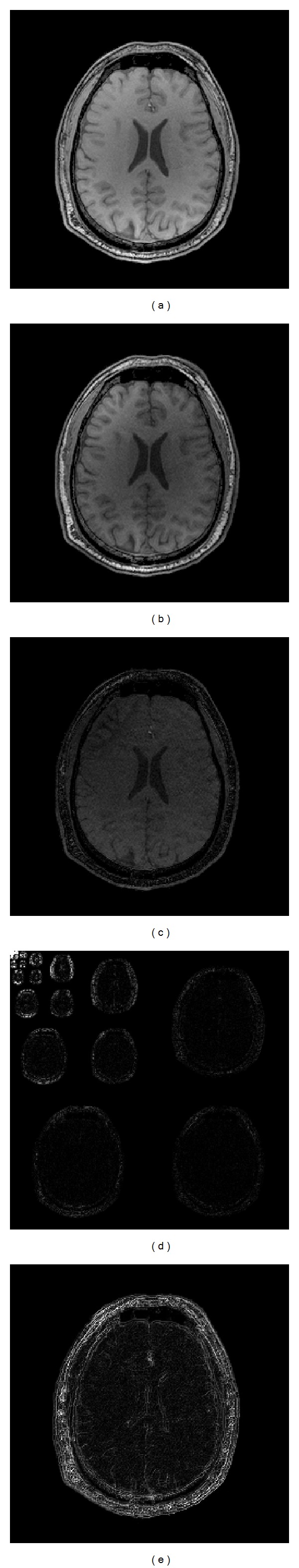
The difference image (c) between the reference image (a) and the target image (b); the wavelet decomposition coefficients (d) and discrete gradients (e) of (c).

**Figure 2:**
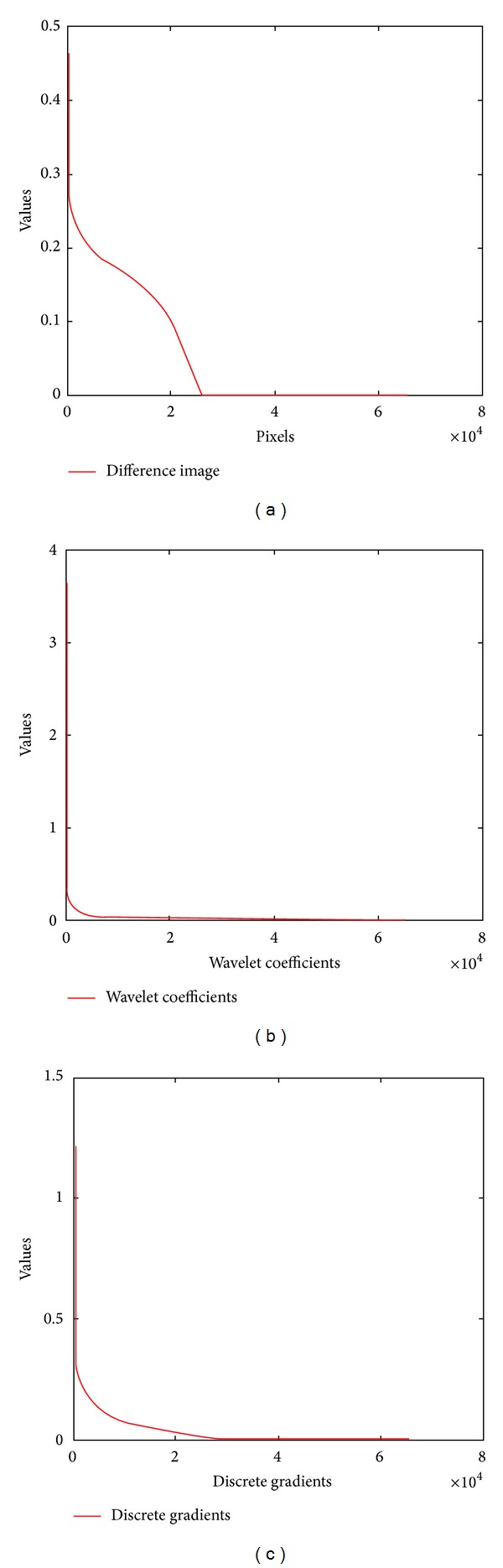
The sparsity of the difference image in the pixel domain (a), wavelet (b), and gradient (c) domains.

*(A) The Influence of Contrast Changes on the Sparsity.* Contrast changes will degrade the sparsity of the difference image in pixel domain. Figures 1(a) and 1(b) are the reference image and the target image, respectively, which are chosen from an MRI sequence of one patient. The MRI data is provided by Professor N. Schuff at the UCSF School of Medicine.  Figure 1(c) shows the difference image. We can see that contrast changes exist between Figures 1(a) and 1(b), which degrade the sparsity of the difference image in pixel domain.  Figure 1(d) shows the wavelet decomposition coefficients of the difference image, and Figure 1(e) is the result from performing second-order TGV operator on the difference image. We also sort pixel values, wavelet decomposition coefficients, and discrete gradients of the difference image in descending order and plot the decay curves in Figure 2. It can be observed that curves in Figures 2(b) and 2(c) descend more steeply than Figure 2(a), which demonstrate that the wavelet coefficients and discrete gradients are exponential damping faster. They demonstrate that the difference image presents much better sparsity (or compressibility) in wavelet transform and gradient domains than in pixel domain. Based on this fact, constraining the sparsity of the difference image in these two domains can effectively weaken the influence of the contrast changes on the sparsity. Therefore, we do not need to estimate the contrast changes.

**Figure 3:**
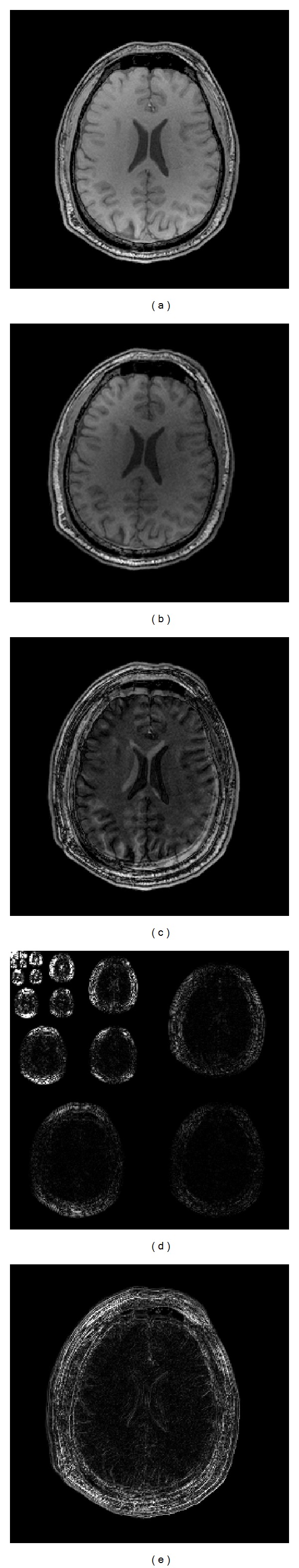
The difference image (c) between the reference image (a) and the target image (b); the wavelet decomposition coefficients (d) and discrete gradients (e) of (c).

*(B) The Influence of Motion Effects on the Sparsity.* Now we analyze the influence of the motion effects on the sparsity of the difference image in pixel, wavelet transform, and gradient domains.  Figure 3(a) is the reference image, and the target image is shown in Figure 3(b) which is obtained by deforming the image in Figure 1(b) through a random affine transformation.  Figure 3(c) is the difference image.  Figure 3(d) displays the wavelet decomposition coefficients and Figure 3(e) is the result of performing second-order TGV on Figure 3(c). It can be observed that motion effects reduce the sparsity of the difference image in all three domains but most severely in pixel domain. This can be proved by the amount of energy change confined in top 5% of pixels, wavelet coefficients, and discrete gradients. The values are 19.37%, 2.76%, and 3.15%, respectively, which indicate that the sparsity of the wavelet coefficients and discrete gradients is relatively not sensitive to the motion effects. To maintain the sparsity of the difference image, performing a rough motion compensation is necessary. Therefore, we propose to estimate the global motion effects by solving (4) before reconstruction. The motion compensation is not involved in iterative reconstruction procedure.

*(C) TGV.* TGV is a seminorm defined in a Banach space. It generalizes TV and is more suitable to describe intensity variation in smooth regions owing to the property that each function of bounded variation admits a finite TGV value. Furthermore, TGV has translation invariance and rotational invariance which meet the requirement that images are measured independently from the actual viewpoint.

Besides the first-order derivative, TGV involves higher-order derivatives to measure image characteristics. Reconstruction with TGV is capable of preserving shape edges without causing staircasing artifacts. Throughout this paper, we use the discrete TGV of second-order formulated in [21]:

(7) $$\begin{array}{c} \text{TG}{{\text{V}}_{\alpha }}^{2}\left(I_{d}\right)=\underset{v}{\min}\alpha_{1}{||\nabla I_{d}-v||}_{1}+\alpha_{0}{||ɛ(v)||}_{1}, \end{array}$$

where

(8) $$\begin{array}{c} ɛ\left(v\right)=\frac{1}{2}\left(\nabla v+\nabla v^{T}\right) \end{array}$$

denotes the symmetrized derivative and ∇ is the first-order differential operator. The positive parameters *α*_0_ and *α*_1_ control the balance between the first and second derivatives. Usually, selecting *α*_0_ as 2*α*_1_ is suitable for most applications. In the proposed method, the discrete gradients yielded by second-order TGV are calculated through solving the minimization problem in (7).

### 2.3. Algorithm and Properties

*(A) Algorithm.* In the proposed reconstruction model (5), there are two nonsmooth regularization terms. We employ FCSA to solve this composite regularization problem. The recently presented FCSA combines the variable and operator splitting techniques and decomposes the complex regularization problem into two simpler subproblems. Furthermore, thanks to the low computational complexity and strong convergence properties, FCSA exhibits its superior performance for MR image reconstruction.

Algorithm 1 describes the algorithm based on FCSA for the difference image reconstruction problem given in (5).

The target image can be obtained as ${\hat{I}}_{t}={\tilde{I}}_{r}+{\hat{I}}_{d}$.

An introduction for the notations and functions presented in Algorithm 1 is as follows.

 (1) ∇*f*(**r** ^*k*^) denotes the gradient of the function *f* at the point **r** ^*k*^. In our paper,
  (9) $$\begin{array}{c} f\left(r^{k}\right)=\;{||d_{t}-F_{u}({\tilde{I}}_{r}+r^{k})||}_{2}^{2}, \end{array}$$
 so
  (10) $$\begin{array}{c} \nabla f\left(r^{k}\right)=2{F_{u}}^{T}\left(F_{u}r^{k}+F_{u}{\tilde{I}}_{r}-d_{t}\right). \end{array}$$
 (2) Given a continuous convex function *g*(*x*), its proximal map is
  (11) $$\begin{array}{c} {\text{prox}}_{\rho }\left(g\right)\left(x\right)=\arg\underset{u}{\min}\left\{g\left(u\right)+\frac{1}{2\rho }{||u-x||}_{2}^{2}\right\}, \end{array}$$
 where scale *ρ* > 0. Therefore, the TGV regularization subproblem is formed as
  (12) I^d1=proxρ(2TGVα2(Id))(Ig)=argmin⁡Id{2TGVα2(Id)+12ρ||Id−Ig||22}.
 Here, the first-order primal-dual algorithm [28] is used to solve this optimization problem, which actually is a denoising problem. The implementing procedure is described in detail in [21].
 (3) The function *x* = project(*x*, [*l*, *u*]) is defined as
  (13) $$\begin{array}{c} x=\text{project}\left(x,\left[l,u\right]\right)=\{\begin{array}{cc} x & l\leq x\leq u\\ l & x<l\\ u & x>u, \end{array} \end{array}$$
 which is used to limit *x* to the range of [*l*, *u*].

*(B) Convergence.* In the step of applying primal-dual algorithm to solve TGV regularization subproblem based on formulation equation (12), the convex-concave saddle-point problem is obtained as follows:

(14) min⁡Id,v max⁡p,q12ρ||Id−Ig||22+〈∇Id−v,p〉 +〈ɛ(v),q〉−δP(p)−δQ(q),

where *G*(**I**_**d**_) = (1/2*ρ*)||**I**_**d**_−**I**_**g**_||_2_^2^ is convex, while functions F*(**p**) = *δ*_*P*_(**p**) and F*(**q**) = *δ*_*Q*_(**q**) are nonconvex. According to Theorem  2 in [28], *O*(1/*N* ^2^) convergence can be guaranteed.

Furthermore, the main algorithm used to reconstruct the difference image is FCSA, whose convergence has been proved in [24]. Therefore, the convergence of Algorithm 1 can be ensured.

*(C) Computational Complexity*. At each iteration, step 3 costs *O*(*mn*log⁡(*mn*)) (where *m* × *n* is the size of the image) since $f\left(r^{k}\right)={||d_{t}-F_{u}({\tilde{I}}_{r}+r^{k})||}_{2}^{2}$. Step 4  ${\hat{I}}_{d1}=\text{pro}{\text{x}}_{\rho }(2\text{TG}{{\text{V}}_{\alpha }}^{2}(I_{d}))(I_{g})$ implemented by primal-dual algorithm costs *O*(*mn*). Similar to the analysis in [24], step 5 has a close form solution with the computational complexity of *O*(*mn*log⁡(*mn*)). In addition, steps 6 and 9 involve additions of vectors, so they result in the computational cost of *O*(*mn*). Step 8 costs *O*(1), in which there is only addition of scalars. And step 7 has the complexity of *O*(*mn*). Consequently the total computational complexity of each iteration in Algorithm 1 is *O*(*mn*log⁡(*mn*)).

## 3. Results

The experiments were conducted on a 2.6 GHz PC with a 32-bit processor having 2 GB RAM. We did the simulations in MATLAB 2011b environment running on Windows XP.

**Figure 4:**
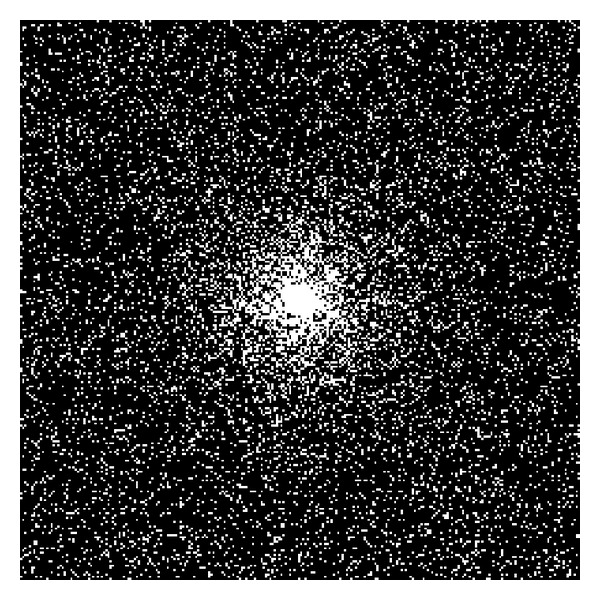
The variable density sampling pattern with 15% sampling rate.

We used the variable density undersampling pattern which is possible to remove the aliasing interference without degrading the image quality [7]. Such sampling pattern is usually used in *k*_*y*_-*k*_*z*_ plane for 3D imaging.  Figure 4 shows such a pattern with 15% sampling ratio. Similar to prior work on CS-MRI [9, 18], the MR images used in the experiments were reconstructed using the fully sampled data, and simulated *k*-space data was generated by undersampling the 2D discrete Fourier transform of the images according to the sampling pattern shown in Figure 4 throughout this paper.

To estimate the performance of the proposed method, we performed the experiments on three sets of data and compared the experimental results of the proposed method with other three methods: (1) sparse-MRI presented by Lustig et al. in [7], (2) referenceless method exploiting wavelet sparsity and TGV constraints (RL-WSTGV), which is formulated as

(15) $$\begin{array}{c} {\hat{I}}_{t}=\arg\underset{I_{t}}{\min}{||d_{t}-F_{u}I_{t}||}_{2}^{2}+\lambda {||\Psi I_{t}||}_{1}+\text{TG}{{\text{V}}_{\alpha }}^{2}\left(I_{t}\right), \end{array}$$

and (3) the reference-driven method in [18], which was renamed as sparse-Id hereafter. Sparse-Id improves the sparsity of the difference image in pixel domain by estimating a uniform contrast change and compensating the motion effects in each iterative step of the reconstruction. For all experiments, we set *λ* = 1*e* − 3 and *α*_0_ = 2*α*_1_ = 5*e* − 3 in the proposed method, which yielded good reconstruction results. By default, the parameter *ρ* of proximal map function in FCSA was set to 1. The number of iterations for FCSA was 100.

**Figure 5:**
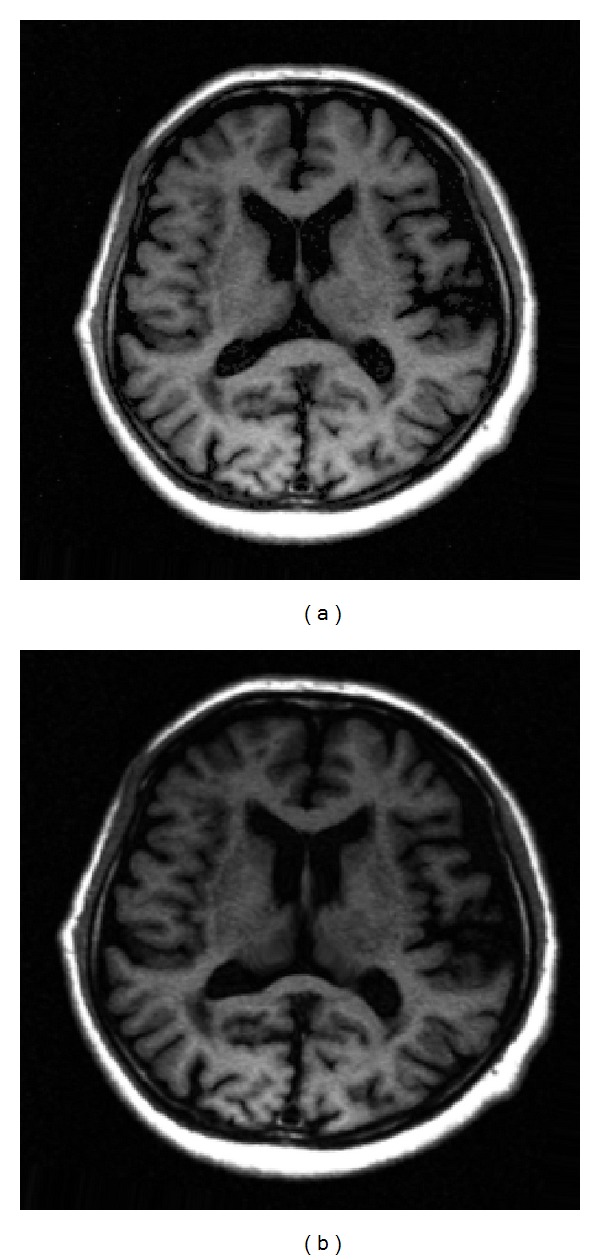
The reference image (a) and the target image (b).

**Figure 6:**
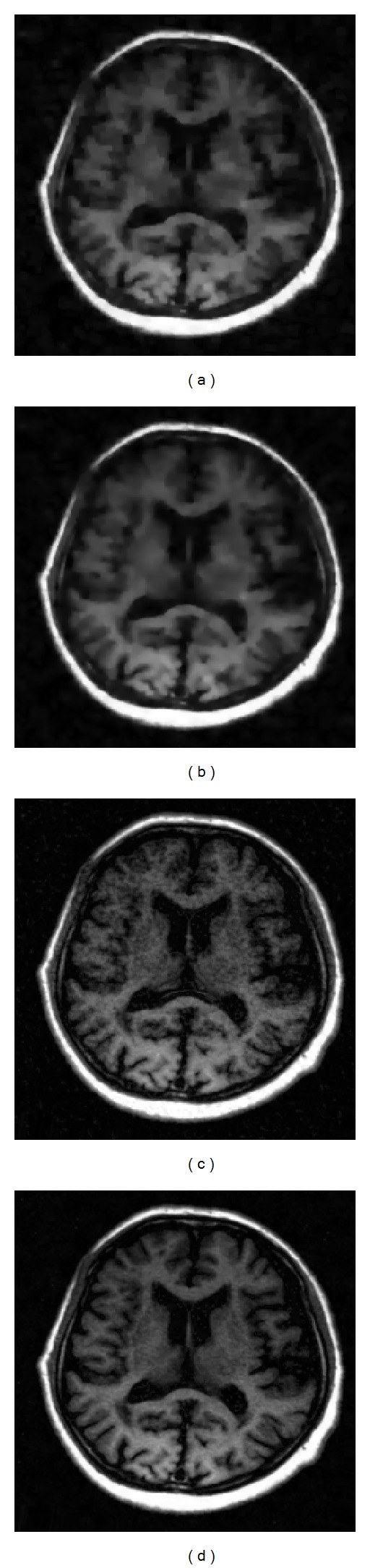
Reconstruction results with 15% of the fully sampled data by sparse-MRI (a), RL-WSTGV (b), sparse-Id (c), and the proposed method (d).

The reference image in Figure 5(a) was with the resolution of 256 × 256, which was acquired on Neusoft Philips scanner 0.35T (superstar LVSM-P035), SE sequence. Imaging parameters were as follows: TR = 1640.1 ms, TE = 15 ms, slice thickness = 6.0 mm, flip angle = 90°, and the field of view (FOV) was 210 × 210 mm^2^. We created the target image in Figure 5(b) based on Figure 5(a) to simulate the situation when obvious nonuniform contrast changes and obvious global motion effects exist in the target image.  Figure 6 shows the images reconstructed by sparse-MRI, RL-WSTGV, sparse-Id, and the proposed method from 15% undersampled measurements. The relative errors and PSNR values are shown in Table 1. As seen from the simulation results in Figure 6 and Table 1, using the reference image can significantly improve the quality of the reconstructed image at a low sampling ratio and the proposed method outperforms sparse-Id.

**Figure 7:**
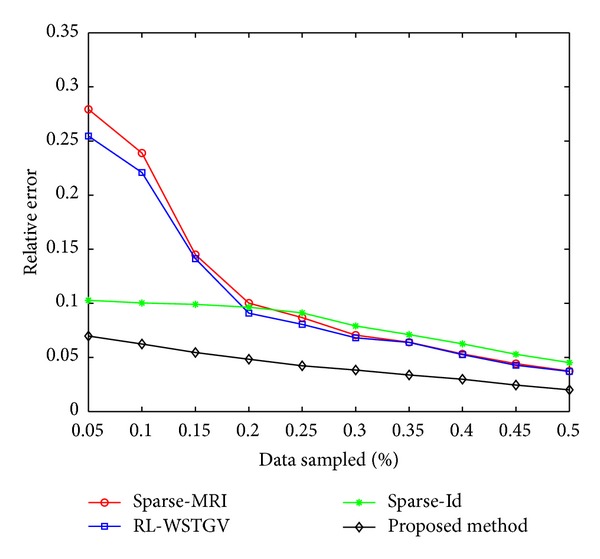
Relative errors of reconstructed images by different methods.

Figure 7 compares the relative errors of reconstructions by all the methods. The curves characterize the relationship between the accuracy of reconstruction and the percentage of data acquired. The proposed method exhibits better reconstruction performance than the other three methods under any sampling ratio.

**Figure 8:**
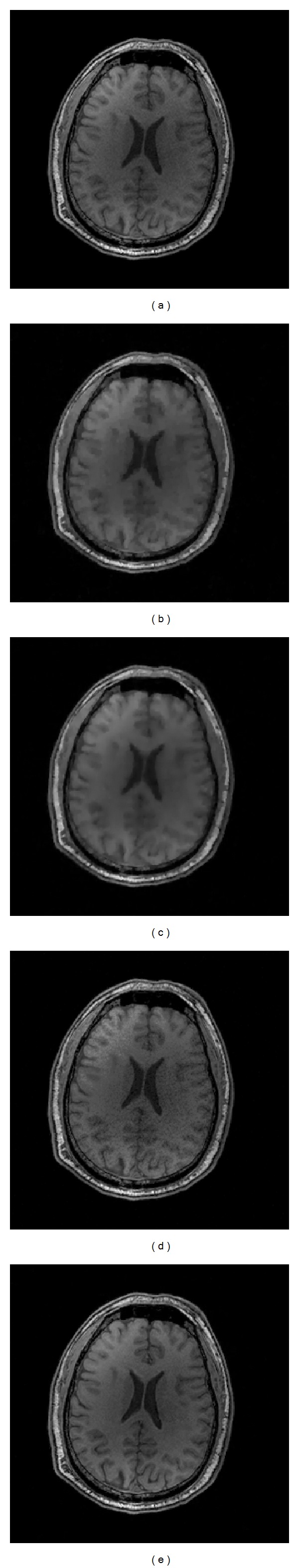
Reconstruction results for the target image (a) with 20% of the fully sampled data by sparse-MRI (b), RL-WSTGV (c), sparse-Id (d) and the proposed method (e).

**Figure 9:**
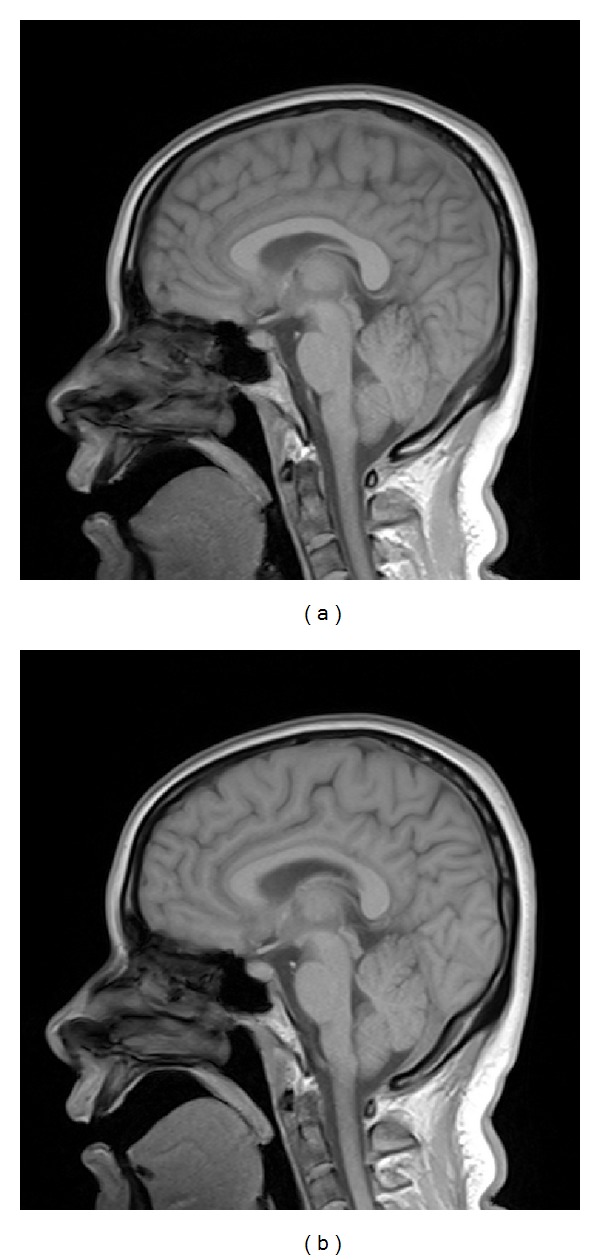
The reference image (a) and the target image (b).

**Figure 10:**
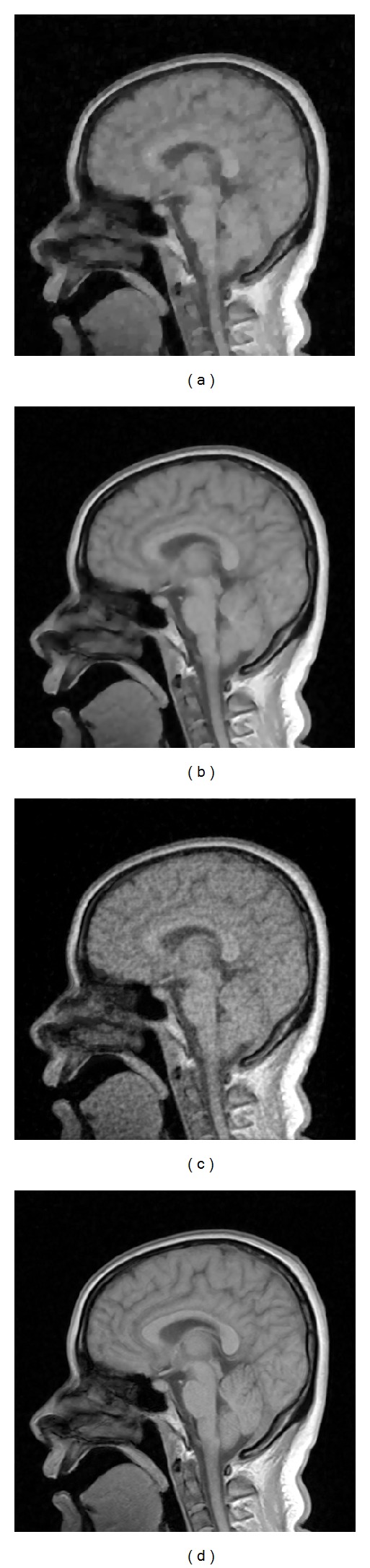
Reconstruction results with 15% of the fully sampled data by sparse-MRI (a), RL-WSTGV (b), sparse-Id (c), and the proposed method (d).

The second experiment was conducted on the MR images shown in Figures 3(a)-3(b). The reconstruction results are given in Figure 8. The third experiment was based on the MR images from one patient shown in Figures 9(a)-9(b), which were acquired on Siemens scanner 3T, GR sequence. Imaging parameters were as follows: TR = 250 ms, TE = 2.5 ms, slice thickness = 5.0 mm, flip angle = 70°, FOV = 220 × 220 mm^2^. The MR images were of size 512 × 512. Figures 10(a)–10(d) show the reconstruction results.

As illustrated by the reconstruction results of these two experiments, we see that the images reconstructed by sparse-MRI and RL-WSTGV lose some details and structures, while RL-WSTGV outperforms sparse-MRI slightly due to the superiority of TGV. The images reconstructed by sparse-Id introduce some artifacts. Our method obtains the best reconstruction results.

## 4. Discussion

### 4.1. Parameter Evaluation

**Figure 11:**
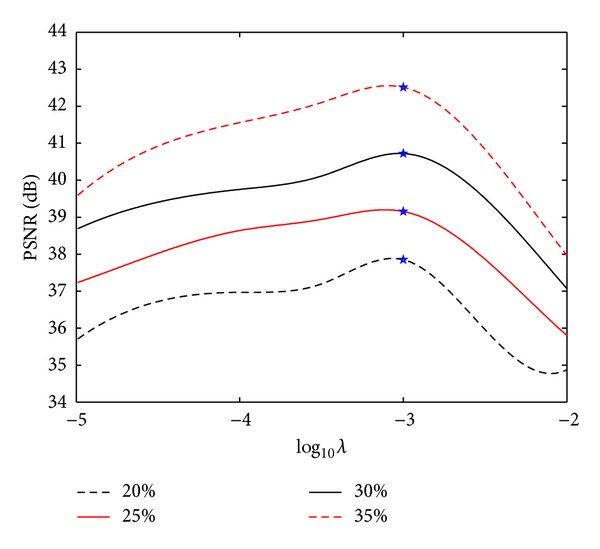
PSNRs versus regularization parameter *λ* for the reconstruction under different sampling ratios (Fix *α*_0_ = 0.005). Asterisks indicate the selected *λ*-values.

**Figure 12:**
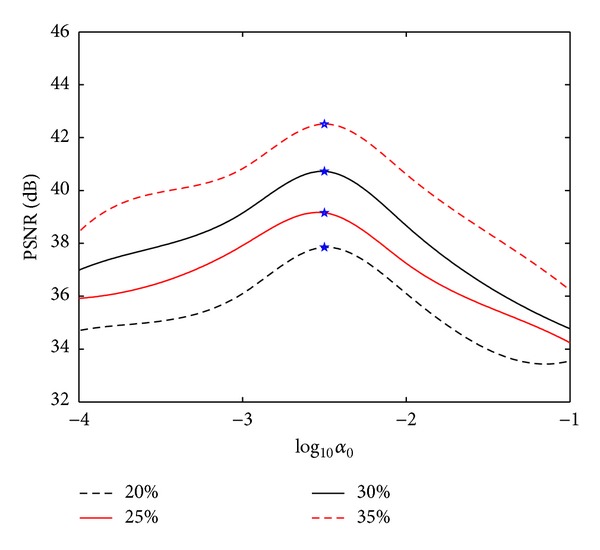
PSNRs versus regularization parameter *α*_0_ for the reconstruction under different sampling ratios (Fix *λ* = 0.001). Asterisks indicate the selected *α*_0_-values.

Figures 11-12 show the plots of PSNR values as a function of the regularization parameters *λ* and *α*_0_ for the reconstruction of the target image shown in Figure 5 under four different sampling ratios. The selected parameter values are marked with asterisks. Seen from the curves, optimal regularization parameters in the proposed method under different sampling ratios are identical, which means that the parameters are robust to the sampling ratio.

### 4.2. Robustness Analysis

**Figure 13:**
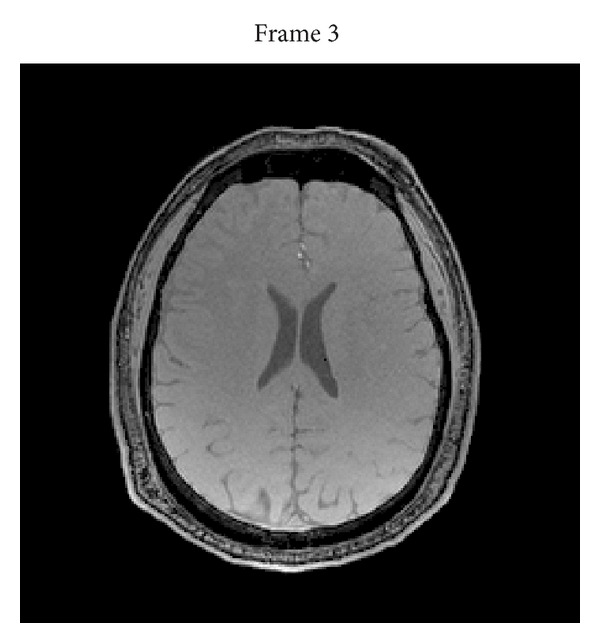
The reference image.

**Figure 14:**
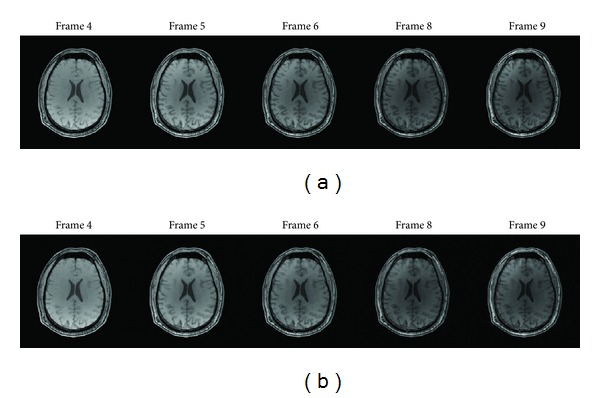
The reconstruction results of an MRI sequence with 20% of the fully sampled data. (a) Target images to be reconstructed. (b) Corresponding reconstructions by the proposed method.

We first discuss the robustness of the proposed method to contrast changes, which is presented by using an MRI sequence of one patient. The sequence is provided by Professor N. Schuff at the UCSF School of Medicine and contains 9 frames. There are only contrast changes between different scans. From Frame 1 to 9, the pixel intensity decreases. Choose Frame 3 as the reference image shown in Figure 13 and reconstruct other frames by employing the proposed method.  Figure 14 gives the reconstruction results for several frames, which displays acceptable visual quality. In particular for Frame 9, although the contrast difference is quite severe, the proposed method still achieves reconstruction successfully.

Generally, the motion of major parts of the anatomy under the scanner is slow in practical application. Thus, big global motions hardly happen between several consecutive time frames. Furthermore, in (4), estimations of global motion parameters mainly lie in the measurements and have nothing to do with the regularization model. Local deformations contribute to the difference image and also can be considered as local contrast changes. Therefore, the robustness of the proposed method to motions is not difficult to be understood.

## 5. Conclusion

This paper has proposed a feasible reference-driven reconstruction method for MR images. We exploit the sparsity of the difference image in wavelet transform and gradient domains to decrease the sampling ratio. In addition, TGV is further introduced to yield sparse discrete gradients and avoid staircasing effects. No contrast changes are needed to be estimated and global motion compensation is done only at the first step of the reconstruction. The proposed method improves the reconstruction quality compared with the conventional referenceless CS-MRI and pixel-sparsity based reference-driven reconstruction methods. We expect the proposed method to be useful for various applications such as interventional imaging and dynamic contrast-enhanced imaging.

## Figures and Tables

**Algorithm 1:**
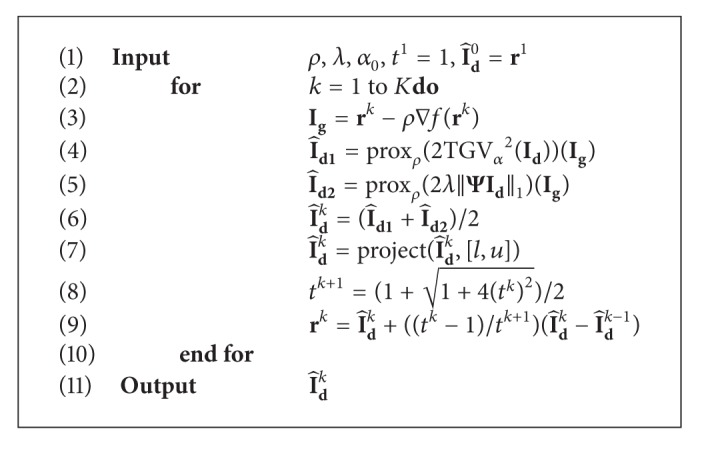
The difference image reconstruction by FCSA.

**Table 1:** Relative errors and PSNR values of reconstructions by different methods under 15% sampling ratio.

Methods	Sparse-MRI	RL-WSTGV	Sparse-Id	Proposed method
Relative error (%)	14.50	14.13	9.91	6.35
PSNR (dB)	26.8336	28.1251	31.1697	35.0748
